# The rural - urban divide in ambulatory care of gastrointestinal diseases in Taiwan

**DOI:** 10.1186/1472-698X-13-15

**Published:** 2013-03-08

**Authors:** Yi-Hsuan Lin, Yen-Han Tseng, Yi-Chun Chen, Ming-Hwai Lin, Li-Fang Chou, Tzeng-Ji Chen, Shinn-Jang Hwang

**Affiliations:** 1 Department of Family Medicine, Kaohsiung Veterans General Hospital Pingtung Branch, Pingtung, No. 1. Anping Lane 1, Jausheng Rd., Pingtung, Neipu Shiang, 91245, Taiwan; 2 Respiratory Therapy Department, Taipei Veterans General Hospital, Taipei, No. 201, Sec. 2, Shipai Rd., Beitou District, Taipei City, Taiwan; 3 Department of Family Medicine, Taitung Veterans Hospital, Taitung, No. 1000, Gengsheng Rd., Taitung City, Taitung County, 95050, Taiwan; 4 Department of Family Medicine, Taipei Veterans General Hospital, Taipei, No. 201, Sec. 2, Shipai Rd., Beitou District, Taipei City 11217, Taiwan; 5 School of Medicine, National Yang-Ming University, Taipei, No. 155, Sec. 2, Linong Street, Taipei 112, Taiwan; 6 Department of Public Finance, National Chengchi University, Taipei, No. 64, Sec. 2, ZhiNan Rd., Wenshan District, Taipei City, 11605, Taiwan

**Keywords:** Healthcare disparities, Gastroenterology, Rural health services, Utilization

## Abstract

**Background:**

The utilization of medical care for gastrointestinal diseases increased over the past decade worldwide. The aim of the study was to investigate the difference between rural and urban patients in seeking medical service for gastrointestinal diseases at ambulatory sector in Taiwan.

**Methods:**

From the one-million-people cohort datasets of the National Health Insurance Research Database, the utilization of ambulatory visits for gastrointestinal diseases in 2009 was analyzed. Rural patients were compared with urban and suburban patients as to diagnosis, locality of visits and choice of specialists.

**Results:**

Among 295,056 patients who had ambulatory visits for gastrointestinal diseases in 2009, rural patients sought medical care for gastrointestinal diseases more frequently than urban and suburban patients (1.60 ± 3.90 *vs.* 1.17 ± 3.02 and 1.39 ± 3.47). 83.4% of rural patients with gastrointestinal diseases were treated by non-gastroenterologists in rural areas. Rural people had lower accessibility of specialist care, especially for hepatitis, esophageal disorders and gastroduodenal ulcer.

**Conclusion:**

The rural–urban disparity of medical care for gastrointestinal diseases in Taiwan highlighted the importance of the well communication between rural physicians and gastroenterologists. Besides the establishment of the referral system, the medical teleconsultation system and the arrangement of specialist outreach clinics in rural areas might be helpful.

## Background

With the rapid development of gastroenterological science, the utilization of medical care for gastrointestinal (GI) diseases increased over the past decade worldwide [1-3]. The utilization of medical care for GI diseases might be influence by the predisposing factors (e.g. the demographics, social structure, occupation, race), enabling factors (e.g. income, insurance coverage, family resources, community resources) and need (e.g. the perceived need, clinically evaluated need) [4]. Besides, the health care system (e.g. health policy, resources, organization) and consumer satisfaction also played a role [5,6]. In the modern medical environment with networked collaboration, gastroenterologists as subspecialists are unlikely to treat all patients with GI diseases. Such a phenomenon can be also observed in other specialties [7,8]. Apart from the factor at the supply side, the socioeconomic and racial differences at the demand side resulted in the disparities of utilization of specialist care [9,10]. Additionally, geography also plays another important role. According to a study in Spain, the referral rate to the GI outpatient clinic was significantly higher in patients from urban areas than in those from rural areas [11]. On the other side, rural patients might have the risk of underutilization of adequate health care resources. Because the relevant literature is scarce, it deserves investigations whether the rural–urban disparities in GI care prevail universally.

The aim of our study was to conduct a nationwide, population-based study to investigate the difference between rural and urban patients in seeking medical service for GI diseases at ambulatory sector in Taiwan. Besides epidemiological measurements, our analyses of locations and specialties would be further stratified by disease group. Our results might also contribute to policy planning of health care resources as well as curricular design of continuing medical education for physicians practicing in rural areas.

## Methods

### Data sources

The data were obtained from the National Health Insurance Research Database (NHIRD) in Taiwan. The National Health Insurance started in 1995 and enrolled 99% of 23 million residents in Taiwan. The monthly claims by the contracted health care facilities are processed electronically and later aggregated to form the NHIRD for research use [12,13]. We obtained the dataset of 1,000,000 people in NHIRD (Longitudinal Health Insurance Database 2005: LHID2005). This cohort dataset contained the claims data of 1,000,000 people randomly sampled from all National Health Insurance beneficiaries in 2005. According to the NHIRD, there was no significant difference in age and sex distribution between the beneficiaries in the LHID2005 and the whole beneficiaries in the National Health Insurance [14]. All claims data belonging to the cohort in the subsequent years were also extracted to form the specific dataset suitable for longitudinal follow-up analyses.

### Study design

From the cohort dataset, we analyzed only data of ambulatory visits, excluding those to dental clinics, in 2009. The major diagnosis in each visit was used to identify the patients who sought medical help for GI problems. The diagnostic coding in the NHIRD was based on the International Classification of Diseases, Clinical Modification, version 9 (ICD-9-CM). To group diagnoses, we adopted the Clinical Classifications Software (CCS) developed by Agency for Healthcare Research and Quality, Rockville in the USA [15]. In the CCS, all diagnoses in the ICD-9-CM were categorized into a small number of clinically meaningful categories. In the current study, the GI diseases were defined as those diagnoses under the single-level CCS categories 6, 12–18, 120, 135, 138–155, 214, 250, and 251. Among these categories, three were collective ones with a broader spectrum of disorders not categorized elsewhere. CCS 155 (other GI disorders) included constipation, dysphagia, irritable colon, celiac disease, functional diarrhea, intestinal fistula, perforation of intestine, etc. CCS 141 (other disorder of stomach and duodenum) included gastroparesis, gastric diverticulum, fistula, acquired hypertrophic pyloric stenosis, etc. CCS 151 (other liver diseases) included liver abscess, cirrhosis, portal hypertension, hepatic coma, ascites, jaundice, hepatomegaly, liver transplant status, etc.

From the claim record of each visit, we could identify the physician’s specialty. The identification number of the clinic in each visit could be linked to the town where the clinic was located. According to the definition of urbanization published by Taiwan’s National Health Research Institutes [16], all 365 townships in Taiwan were classified into 7 levels based on the following variables: population density (people/km^2^), population ratio of people with college or above educational levels, population ratio of elder people over 65 years old, population ratio of people of agriculture workers and the number of physicians per 100,000 people. To categorize the location in the current study, we operationally defined townships of levels 5–7 as rural, levels 3–4 as suburban and levels 1–2 as urban.

In the NHIRD, the information about each beneficiary’s residence was not available. To categorize the patients into rural, suburban and urban groups, we devised an algorithm on the basis of the location of physician clinics and local community hospitals in which the patients most frequently sought medical visits (not limited to GI diagnoses) during 2007 to 2009. If a patient sought medical visits at rural clinics and rural local community hospitals for more than or equal to 60% of such visits, she or he was deemed as a rural resident in our study. Suburban and urban residents were defined by the same rationale. If none of the above conditions existed, the patient was deemed as a migratory resident.

Other patient features under analysis included gender, age, GI diagnosis, as well as location of clinics, type of clinics (GI clinics or non-GI clinics) and number of visits for GI diseases. Groups of rural, suburban and urban patients would be compared.

### Statistical methods

Data extraction and computation were performed with the Perl programming language, version 5.12.1 [17]. SPSS software (version 17) was used for statistical analysis. Besides the descriptive statistics, we used Pearson’s χ^2^ tests for categorical variables and ANOVA test for continuous variables. A *p*-value <0.05 (two-tailed) was considered as statistically significant.

## Results

In 2009, only 879,240 beneficiaries in the cohort dataset had ambulatory visit records and 295,056 (33.5%) people had visits for GI diseases. Among the latter patient group, only 5.2% (n = 15,340) belonged to rural residents while about two-thirds (n = 193,705) belonged to urban, 15.6% (n = 46,083) to suburban and 13.5% (n = 39,928) to migratory residents.

Of all rural residents, 40.4% had visits for GI diseases during the year, higher than 38.2% in suburban residents and 35.6% in urban residents (Table 1). The mean number of visits for GI diseases by each rural resident in 2009 was also higher than those by each suburban and urban resident (1.60 ± 3.90 *vs.* 1.39 ± 3.47 and 1.17 ± 3.02, *p* < 0.001). In each resident group of different urbanization, female were more likely to have visits for GI diseases than males (*p* < 0.001) and people older than 65 years also sought medical help for GI diseases more frequently than younger people (*p* < 0.001).

**Table 1:** The rural–urban divide in the number of patients and number of visits with GI diseases, stratified by gender and age

	**Rural**			**Suburban**			**Urban**		
	**Beneficiaries**	**Pat. with GI disease (%)**	**No. of visits with GI disease per beneficiary (mean ± SD)**	**Beneficiaries**	**Pat. with GI disease (%)**	**No. of visits with GI disease per beneficiary (mean ± SD)**	**Beneficiaries**	**Pat. with GI disease (%)**	**No. of visits with GI disease per beneficiary (mean ± SD)**
Female									
0-17	3654	1357 (37.1)	0.79 ± 1.70	11886	4408 (37.1)	0.82 ± 1.76	44476	16150 (36.3)	0.79 ± 1.71
18-39	3261	1329 (40.8)	1.27 ± 2.91	16293	6577 (40.4)	1.18 ± 2.62	110506	42228 (38.2)	1.05 ± 2.46
40-64	6664	3009 (45.2)	1.77 ± 3.69	21141	8926 (42.2)	1.64 ± 3.65	103375	39711 (38.4)	1.43 ± 3.46
> = 65	4464	2243 (50.2)	2.48 ± 5.04	10493	4846 (46.2)	2.26 ± 4.72	29858	12907 (43.2)	2.07 ± 4.49
Subtotal	18043	7938 (44.0)*	1.66 ± 3.71**	59813	24757 (41.1)*	1.46 ± 3.37**	288215	110996 (38.5)*	1.25 ± 3.05**
Male									
0-17	3971	1449 (36.5)	0.78 ± 1.58	13037	4827 (37.0)	0.78 ± 1.64	48661	17922 (36.8)	0.79 ± 1.74
18-39	4242	1199 (28.3)	0.81 ± 2.57	16399	4456 (27.2)	0.77 ± 2.42	88672	23716 (26.7)	0.70 ± 2.10
40-64	7480	2791 (37.3)	1.77 ± 4.47	21394	7530 (35.2)	1.54 ± 4.02	91968	29655 (32.2)	1.26 ± 3.35
> = 65	4236	1963 (46.3)	2.66 ± 5.58	10007	4513 (45.1)	2.48 ± 5.23	27082	11416 (42.2)	2.24 ± 4.94
Subtotal	19929	7402 (37.1)*	1.56 ± 4.07**	60837	21326 (35.1)*	1.32 ± 3.57**	256383	82709 (32.3)*	1.08 ± 2.99**
Total	37972	15340 (40.4)*	1.6 ± 3.90	120650	46083 (38.2)*	1.39 ± 3.47	544598	193705 (35.6)*	1.17 ± 3.02

* *p *<0.001 of Chi-square test for the no. and percentage of patients with GI diseases among different areas.

**p <0.001 of ANOVA test for the no. of visits with GI disease within the same gender among different areas.

GI: gastrointestinal; Pat: patients; SD: standard deviation.

About four-fifths of rural patients with GI diseases were treated at non-GI clinics in rural areas (83.4%, n = 12,796). When rural patients decided to visit gastroenterologists, urban GI clinics were more frequently chosen than suburban GI clinics (7.9% *vs.* 5.2%, *p* < 0.001). Compared with rural residents, urban and suburban residents were more likely to visit GI clinics (19.0% and 17.9% *vs.* 15.2%, *p* < 0.001) (Table 2).

**Table 2:** Types of clinics visited by patients with GI diseases, stratified by locality

	**Rural patients no (%, n = 15340)**	**Suburban patients no (%, n = 46083)**	**Urban patients no (%, n = 193705)**
Rural areas			
GI clinics	465 (3.0)	74 (0.2)	23 (0.0)
Non-GI clinics	12796 (83.4)	1597 (3.5)	1534 (0.8)
Suburban areas			
GI clinics	791 (5.2)	5232 (11.4)	1205 (0.6)
Non-GI clinics	2059 (13.4)	38793 (84.2)	5341 (2.8)
Urban areas			
GI clinics	1216 (7.9)	3289 (7.1)	35810 (18.5)
Non-GI clinics	2117 (13.8)	6781 (14.7)	173645 (89.6)
All areas			
GI clinics	2333 (15.2)*	8251 (17.9)*	36815 (19.0)*
Non-GI clinics	14637 (95.4)	42914 (93.1)	177209 (91.5)

* *p *<0.001 of Chi-square test for the number and percentage of patients with GI diseases attending GI clinics among different residences.

GI: gastrointestinal.

**Figure 1:**
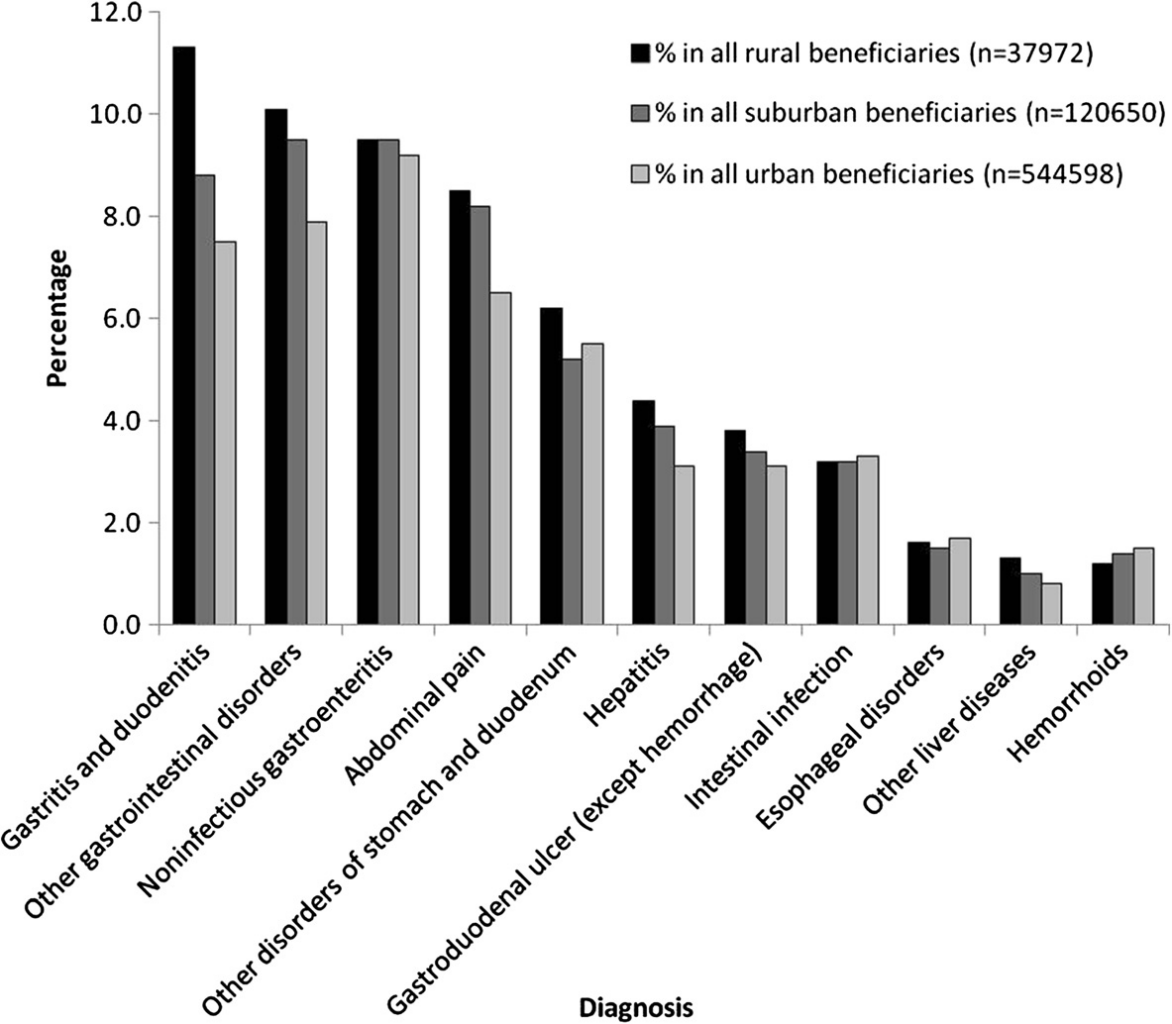
Prevalence of GI diseases, stratified by patient’s residency and disease group.

**Figure 2:**
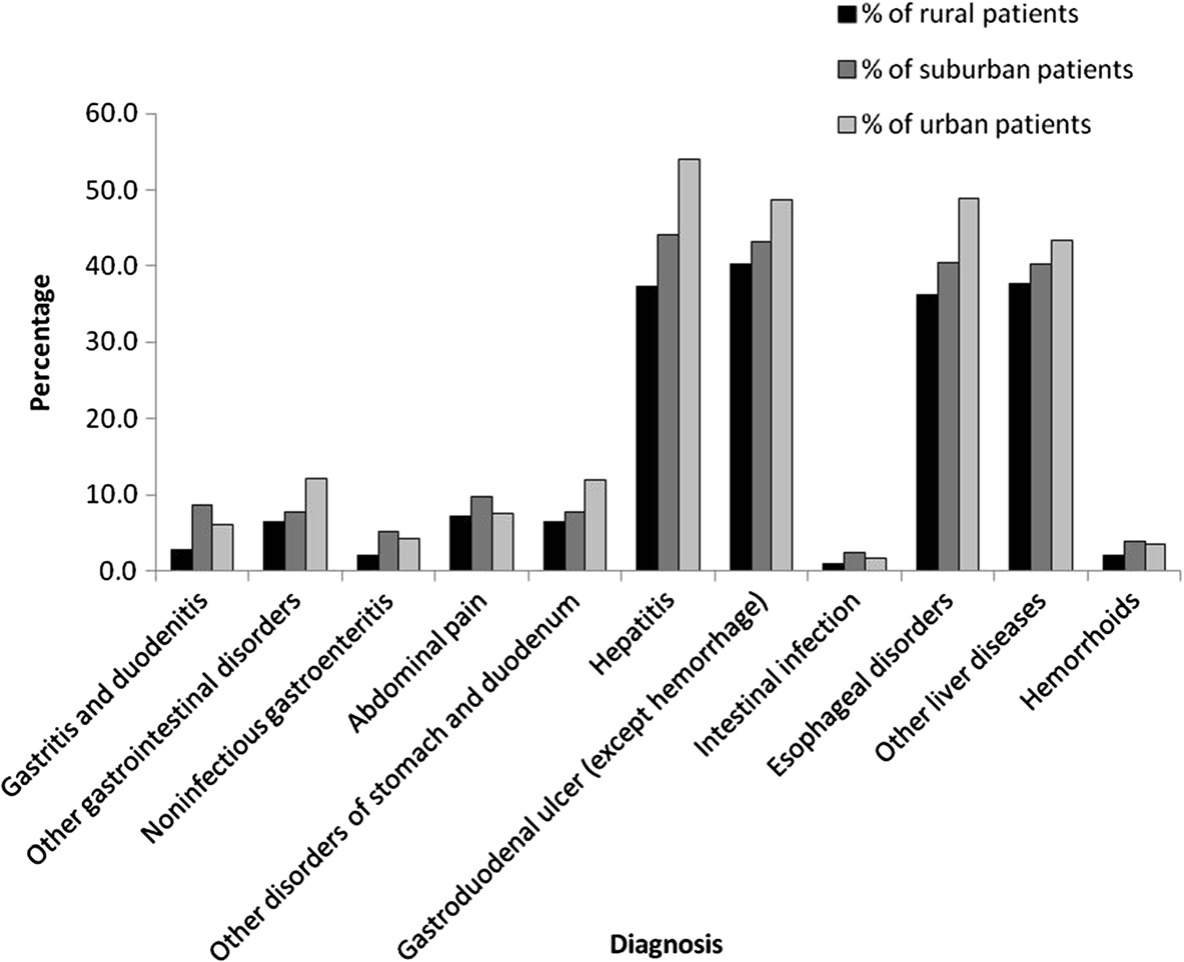
The percentage of GI specialist care, stratified by patient’s residency and disease group.

Patients of different urbanization had similar spectrums of GI diseases with slight discrepancy in ranking order (Table 3). Except intestinal infection, esophageal disorder and hemorrhoids, rural patients had higher prevalences of other kinds of GI diseases than urban patients (Figure 1). However, rural patients were less likely to visit GI clinics than suburban and urban patients in all kinds of GI diseases (Figure 2) and the differences were most striking in hepatitis (37.4% *vs.* 44.2% and 54.0%), esophageal disorder (36.3% *vs.* 40.5% and 48.8%) and gastroduodenal ulcer (40.2% *vs.* 43.1% and 48.7%, all *p* < 0.001).

**Table 3:** Distribution of GI diseases, stratified by patient’s residence

	**Rural**		**Suburban**		**Urban**	
**Disease groups according to clinical classifications software**	**No. of pat.**	**No. of pat. treated at GI clinics (%, n = No. of pat.)**	**No. of pat.**	**No. of pat. treated at GI clinics (%, n = No. of pat.)**	**No. of pat.**	**No. of pat. treated at GI clinics (%, n = No. of pat.)**
Gastritis and duodenitis	4287	117 (2.7)	10669	927 (8.7)	40721	2451 (6.0)
Other gastrointestinal disorders	3844	246 (6.4)	11507	0892 (7.8)	43236	5287 (12.2)
Noninfectious gastroenteritis	3600	76 (2.1)	11417	586 (5.1)	50078	2094 (4.2)
Abdominal pain	3238	229 (7.1)	9860	964 (9.8)	35590	2702 (7.6)
Other disorders of stomach and duodenum	2336	151 (6.5)	6310	484 (7.7)	30098	3624 (12.0)
Hepatitis	1683	630 (37.4)	4685	2070 (44.2)	16671	9000 (54.0)
Gastroduodenal ulcer (except hemorrhage)	1451	583 (40.2)	4141	1784 (43.1)	17004	8274 (48.7)
Intestinal infection	1201	11 (0.9)	3866	91 (2.4)	18165	314 (1.7)
Esophageal disorders	625	227 (36.3)	1862	755 (40.5)	9408	4587 (48.8)
Other liver diseases	492	185 (37.6)	1210	486 (40.2)	4457	1936 (43.4)
Hemorrhoids	456	9 (2.0)	1673	65 (3.9)	7998	283 (3.5)

* Only top 11 diseases were listed.

GI: gastrointestinal.

## Discussion

### Main findings

Our current study of claims analysis revealed that rural residents in Taiwan were more likely to seek medical help for GI diseases than urban and suburban residents. Most rural people with GI problems would visit the non-GI clinics in rural areas. Urban patients were more likely than rural patients to consult gastroenterologists for hepatitis, esophageal disorder and gastroduodenal ulcer.

### Interpretation of findings

The environmental factor might play a role. Although no concrete data had been officially published as to the hygiene situation in rural Taiwan, it was generally deemed that rural residents had less adequate sanitation in water supply and waste disposal. The poor hygiene in rural areas could be associated with infectious GI diseases [18,19].

The lifestyle of rural residents might differ from that of urban residents. For example, rural residents may have higher rates of adult smoking, physical inactivity, and alcohol abuse [20]. Besides, the aging population structure of rural areas could contribute to a higher prevalence of GI diseases. In the USA, a significant number of retirees moved into rural areas and young adults migrated to big cities for job or school, making the population structure of rural America older [21]. Such a phenomenon also existed in Taiwan [22]. The co-morbidities, polypharmacy and possible more drug-drug interactions in the elderly could make this population more vulnerable to suffer from GI problems [23].

Rural residents in Taiwan sought more medical help for gastritis and duodenitis than urban residents. Gastritis and duodenitis might be related to *Helicobacter pylori* infection [24]. The prevalence of *Helicobacter pylori* in Taiwan and China was reported to be higher in rural developing areas than in urban developed ones [25-28]. The transmission of *Helicobacter pylori* in rural areas was more complex and could occur through contaminated food, water or intensive contact between infants and non-parental caretakers [29].

Non-steroidal anti-inflammatory drugs (NSAID) might play another role in the rural–urban divide of visits for GI diseases [24]. In Portugal, the amount of NSAID prescriptions in the ambulatory general practice had been reported to be higher among the elderly and in rural areas [30]. A similar condition might also exist in Taiwan. NSAID might thus cause more frequent occurrences of gastroduodenal ulcer in rural people, what could partly explain the fact that in our current study rural people had more visits for ulcer disease than urban people. However, *Helicobacter pylori* and NSAID-related gastritis and duodenitis usually occur in chronic course. It is uncertain if the course of gastritis or duodenitis was acute or chronic in our study. Further researches are needed in this field.

Although rural patients were more likely to seek medical help for GI disease, most of them chose the non-GI clinics in rural areas. In Taiwan, the diagnoses and management of some specific diseases rely on specialists, but the general management or follow-up post diagnosis could be made by general practitioners. Our result implied that the physicians other than gastroenterologists in rural areas were the main gatekeepers for rural patients when medical services for GI diseases were needed. However, rural physicians might have more barriers to access necessary medical knowledge than non-rural physicians [31]. Physicians in rural areas were also more likely to felt isolated, dissatisfied with job security and frustrated by a lack of cooperation among the major providers of health care [32]. Therefore, the well communication between general practitioners in rural areas and gastroenterologists would be more important than those in non-rural areas. To build an adequate referral system is essential in remote setting [33]. Furthermore, telemedicine would be a solution for bridging geographic access gaps to specialty care [34]. It has also been proposed that the arrangement of specialist outreach clinics in rural areas could increase the accessibility and effectiveness of specialist services and the integration with primary care services [35].

On the other hand, urban patients were more likely than rural patients to consult gastroenterologists for hepatitis, esophageal disorder and gastroduodenal ulcer. One of the causes might be related to regulations of health insurance reimbursement. According to the pharmaceutical benefit scheme of the National Health Insurance in Taiwan [36], proton pump inhibitors can only be prescribed for peptic ulcer and gastroesophageal reflux disease proved by upper gastrointestinal endoscopy. The majority of such procedures are usually performed by gastroenterologists. The prescription of anti-viral agents for viral hepatitis is also limited to gastroenterologists. Because of the higher availability of specialists in urban areas than in rural areas, urban people would be more likely to be diagnosed with above-mentioned diseases.

### Limitations

There were some limitations in our current study. The diagnoses for analysis came from the administrative claims and might be tentative instead of final. Besides, the severity of diseases was hardly available. The reasons of seeking GI care across the rural–urban border could not be truly uncovered either. Some patients seeking a GI specialist at the medical center might be referred from the general practitioners. We couldn’t calculate how many patients were referred to medical centers in the NHIRD. This could cause some bias. However, a formal referral system does not exist in Taiwan. The patients can freely choose the physicians and facilities. Finally, the impact of rural–urban divide on population health was not analyzed. The techniques to identify the outcome of disease management might go beyond the scope of our current study.

### Strengths and impact of the study

Our study was the first nationwide, population-based study to demonstrate the difference of medical seeking behavior between rural and urban patients with GI problems. For those valuable information contained in Taiwan’s NHIRD, to examine the rural–urban disparity of medical service utilization for GI diseases could contribute to the policy making in the future.

## Conclusions

The rural–urban disparity in GI care actually existed in Taiwan and most rural patients sought medical help for GI problems at non-GI clinics. A well-established referral system, telemedicine and even the arrangement of GI specialist outreach clinics to integrate with primary care services in rural areas might help to reduce the geographic gaps for rural patients. Further studies are needed to discover the influence as well as more resolution for the rural–urban divide in GI care.

## Abbreviations

GI: Gastrointestinal; NHIRD: National Health Insurance Research Database; LHID2005: Longitudinal Health Insurance Database 2005; CCS: Clinical Classifications Software.

## Competing interests

The authors declare that they have no competing interests.

## Authors’ contributions

YHL conceived and carried out the study, performed the statistical analysis and drafted the manuscript. YHT and YCC participated in the design of the study and helped to interpret findings. MHL, LFC and SJH participated in the design of the study and helped to perform the statistical analysis. TJC participated in the design and coordination of the study and helped to draft the manuscript. All authors read and approved the final manuscript.

## Pre-publication history

The pre-publication history for this paper can be accessed here:

http://www.biomedcentral.com/1472-698X/13/15/prepub
